# Simulation of a Reverse Electrodialysis–Absorption Refrigeration Integration System for the Efficient Recovery of Low-Grade Waste Heat

**DOI:** 10.3390/membranes15010002

**Published:** 2024-12-24

**Authors:** Xi Wu, Linjing Yan, Xiaojing Zhu, Mingjun Liu

**Affiliations:** 1 Key Laboratory of Ocean Energy Utilization and Energy Conservation of Ministry of Education, School of Energy and Power Engineering, Dalian University of Technology, Dalian 116024, China; 2 Sonyo Refrigeration (Dalian) Co., Ltd., Dalian 116699, China

**Keywords:** absorption refrigeration, reverse electrodialysis, LiBr, energy conversion, waste heat

## Abstract

The absorption refrigeration system (ARS) stands as a remarkable device that is capable of efficiently harnessing low-grade thermal energy and converting it into cooling capacity. The reverse electrodialysis (RED) system harvests the salinity gradient energy embedded in two solutions of different concentrations into electricity. An innovative RED–ARS integration system is proposed that outputs cooling capacity and electric energy, driven by waste heat. In this study, a comprehensive mathematical simulation model of a RED–ARS integration system was established, and an aqueous lithium bromide solution was selected as the working solution. Based on this model, the authors simulated and analyzed the impact of various factors on system performance, including the heat source temperature (90 °C to 130 °C), concentrated solution concentration (3 mol∙L⁻^1^ to 9 mol∙L⁻^1^), dilute solution concentration (0.002 mol∙L⁻^1^ to 0.5 mol∙L⁻^1^), condensing temperature of the dilute solution (50 °C to 70 °C), solution temperature (30 °C to 60 °C) and flow rate (0.4 cm∙s⁻^1^ to 1.3 cm∙s⁻^1^) in the RED stacks, as well as the number of RED stacks. The findings revealed the maximum output power of 934 W, a coefficient of performance (COP) of 0.75, and overall energy efficiency of 33%.

## 1. Introduction

### 1.1. Absorption Refrigeration Technology

Approximately one-third of the energy utilized in actual industrial processes is squandered as waste heat, and 63% of this falls below 100 °C [1,2]. The recovery of this low-grade thermal energy (LTE) holds immense significance in reducing the consumption of fossil fuels and mitigating CO_2_ emissions. Absorption refrigeration systems (ARSs) can efficiently harness LTE and convert it into cooling capacity, exhibiting tremendous promise in the realm of waste heat utilization. The refrigerant–absorbent working pairs cycling inside the ARS usually present minimal to no global warming potential (GWP) and are ozone-layer-friendly. Researchers are diligently developing ARSs tailored to various waste heat sources with different temperatures and qualities, aiming to enhance the overall energy efficiency and reduce the energy consumption and operational costs for enterprises.

Salmi et al. introduced a steady-state thermodynamic model for an absorption refrigeration cycle utilizing water–lithium bromide and ammonia–water working pairs, specifically tailored application in ships [3]. The effects of the generation and evaporation temperatures on the coefficients of performance (COP) of the ARS were investigated. Rubio-Maya et al. optimized an ARS with the ultimate goal of minimizing the annual operating cost. By formulating the optimization problem as a nonlinear programming model, they successfully reduced the complexity of the thermal system [4]. Yan et al. proposed an innovative high-efficiency absorption refrigeration cycle featuring an improved single-effect/double-lift structure and conducted a steady-state simulation study to enhance the utilization efficiency of the available thermal energy from exhaust gas/water [5]. Ebrahimi et al. explored the theoretical feasibility of harnessing heat dissipation from servers and then powered an ARS for the cooling of other servers in the data center [6].

The ARS is mainly driven by thermal energy, whereas the solution pump, data acquisition and control system, etc., necessitate external electrical energy for their operations. The present authors conducted an exhaustive investigation into 610 models of ARS units from five internationally renowned manufacturers from China, the United States, and Japan. The results indicate that the external power supply accounts for approximately 0.2% to 2.5% of the refrigeration capacity of the whole ARS.

### 1.2. Reverse Electrodialysis Method

The escalating energy consumption and substantial carbon emissions make it imperative to develop new power generation technologies that are sustainably utilized, highly efficient, and low-carbon. LTE recovery has increasingly become a pivotal step in establishing sustainable energy systems globally. However, traditional methods, such as the Rankine cycle, demand a heat source temperature exceeding 150 °C [7]. The reverse electrodialysis (RED) heat engine stands out as an effective technology for the exploitation of LTE (60~150 °C) by converting it into salinity gradient energy (SGE), which is then transformed into electricity. When two salt solutions of different concentrations are channeled through the RED stacks, the concentration gradient between the concentrated and dilute solutions prompts the movement of ions across ion exchange membranes (IEMs). This ion flux is governed by the membrane’s selective permeability, specifically the selective transport of cations through the cation exchange membrane (CEM) and anions through the anion exchange membrane (AEM). The ultimate ionic current passing through the membrane undergoes redox reactions at the electrode pair at the two ends of the RED stack, converted into an electrical current when connected with an external load.

In 1954, Pattle first described the concept of the concentration gradient and obtained a power density of 0.2 W∙m^−2^ and an electric potential of 3.1 V from seawater and river water using a membrane stack device consisting of acidic membranes [8]. Veerman et al. proposed a model for the calculation of the voltage based on the membrane mass transfer equation and thermodynamic equation by considering the effects of the spacer network and the non-ideal behavior of IEMs [9]. Kim et al. utilized simulated seawater and river water to feed the RED stack and achieved a power density of 2.4 W∙m^−1^ by using homemade IEMs and mesh gaskets [10]. Daniilidis et al. obtained an output power density of 6 W∙m^−1^ by using a highly concentrated ratio of solutions (5 M/0.1 M) [11]. Wang et al. applied lithium bromide–ethanol–water ternary working solutions to the RED stack, revealing the RED characteristics under different operating conditions [12].

Numerous scholars have conducted research on RED methods in conjunction with solution separation technologies. Micari et al. investigated the utilization of pure and equimolar saline aqueous solutions as feedstocks in a lab-scale RED unit. Their primary objective was to assess the power output of binary mixtures, and the NH_4_Cl-LiCl mixture exhibited the most promising results [13]. Hu et al. combined multi-effect distillation with a RED power generation system. The energy efficiency of the closed-loop system reached 1.01% [14]. An alternative integrated membrane power generation system consisting of membrane distillation and RED was also proposed, with electrical efficiency of up to 1.15% [15]. Liu et al. carried out an analysis on a RED heat engine and found that, when the generation temperature reached 70 °C, the energy efficiency and exergy efficiency could be 2.6% and 19%, respectively [16]. Giacalone et al. analyzed the influence of the thermodynamic properties of unconventional NaCl aqueous solutions on the operation of a closed-loop RED system. Their findings revealed that KAc, CsAc, and LiCl emerged as the most promising salts from their screening process. In addition to its role in electricity generation, hydrogen emerges as an excellent form of energy storage. Zhang et al. proposed and experimentally validated a multi-stage RED stack system, enhancing the efficiency of converting SGE into hydrogen [17]. Wu et al. introduced a novel closed-loop system that converted LTE into hydrogen via RED–air gap diffusion distillation (AGDD) for the first time. This system was powered by the membrane voltage generated by the SGE of KAc solutions [18]. Leng et al. developed an RED–AGDD mathematical model for the simulation of hydrogen production and wastewater purification simultaneously [19].

## 2. Objective and Contributions

Many studies have been conducted on ARS and RED individually, yet there remains a notable gap in RED–ARS integration systems regarding their detailed performance and coupling mechanisms. In the whole system, three working solutions of varying concentrations—a concentrated solution, a dilute solution, and refrigerant in both the solution and refrigerant circuits—undergo continuous circulation and regeneration. Notably, two solutions, differing in concentrations with a salinity gradient, are directly fed to the RED stack, where the SGE of the two solutions is efficiently converted into electrical energy. This RED–ARS integration system not only facilitates the continuous utilization of LTE but also enhances the energy utilization rate, reduces fossil fuel consumption, and achieves the “off-grid” target for the refrigeration system. The unit operates independently of seasonal constraints and is not shut down when the cooling capacity is not required; thus, it could offer dual efficiency in both “heat to cooling” and “heat to electricity” conversion. In this study, a comprehensive mathematical simulation model of a RED–ARS integration system was established. Based on this model, the authors simulated and analyzed the impacts of various factors on system performance, including the heat source temperature, concentrated and dilute solution concentrations, solution temperature, condensing temperature of the dilute solution, flow rate in the RED stacks, and number of RED stacks. The results are beneficial to promote the development of a innovative RED–ARS integration system that outputs cooling capacity and electric energy, driven by SGE.

## 3. System and Mathematical Model Descriptions

### 3.1. System Description

**Figure 1:**
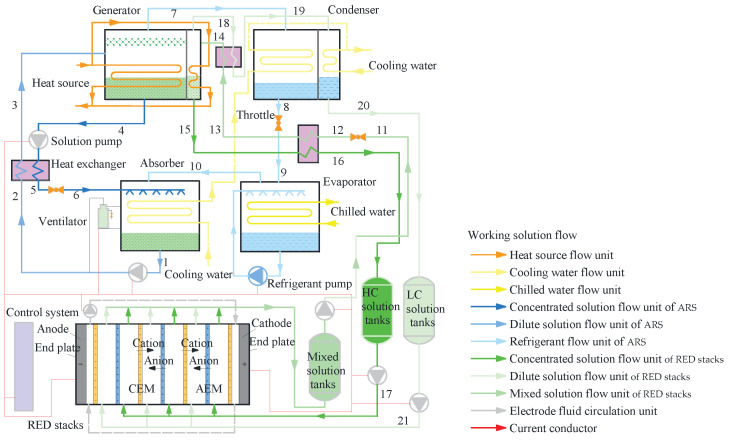
Schematic diagram of RED–ARS integration system.

**Figure 2:**
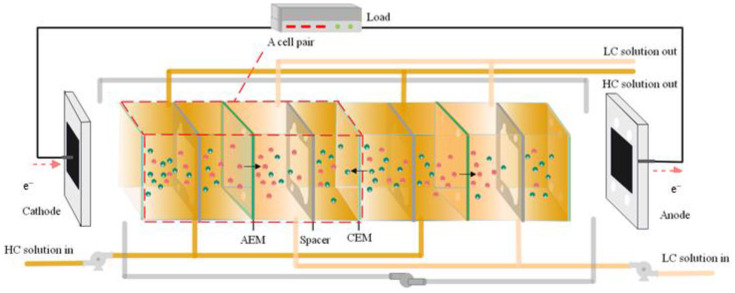
Schematic diagram of a RED stack.

Figure 1 presents a schematic illustration of the interconnected components and relationships within the RED–ARS integration system. This system is composed of two primary subsystems: the ARS subsystem and the RED subsystem. The operational inputs for this system include waste heat harnessed by a generator and electrical energy utilized by solution pumps. The outputs generated by the system consist of refrigerated air by the evaporator and electrical energy outputted by the RED stacks. Figure 2 illustrates the intricate details of a RED stack structure.

The ARS subsystem comprises an ARS generator unit, a condenser, an absorber, an evaporator, and a solution heat exchanger. Meanwhile, the RED subsystem includes a RED generator unit, a condenser, and the RED stacks, which are seamlessly integrated with the RED generator unit.

Low-pressure refrigerant (water) vapor enters the absorber, where it undergoes an exothermic process, being absorbed by the concentrated lithium bromide aqueous solution. The diluted solution in the absorber is subsequently pumped directly into the solution heat exchanger. After being heated, the dilute solution proceeds to the ARS generator unit. In the ARS generator unit, part of the refrigerant is separated from the absorbent through absorbing the waste heat. The refrigerant vapor is then channeled into the condenser, while the produced concentrated solution is depressurized by a throttling device and recycled back to the absorber after releasing heat.

The heat source drives the working solution regeneration process in the RED generation unit exothermically. The effluent solution of the RED stack outlet is recycled back to the RED generation unit for heating until it reaches boiling point. The produced vapor escapes into the RED condensation unit. Consequently, the reserving solution in the RED generation unit is recovered to the concentrated solution and then enters into a solution heat exchanger to dissipate the heat.

The concentrated and diluted solutions are then channeled into their respective flow compartments, which are alternately arranged within the RED stack. After converting the SGE into electrical energy via the RED process, the working solution flows out of the RED stack. The effluent concentrated and dilute solutions are recycled back to the RED generation unit for regeneration. Notably, in the RED subsystem, there are a number of RED stacks connected in series. It is configured in multiple stages, where the concentrated and dilute solutions sequentially flow through each RED stack.

The structural and operational parameters are listed in Table 1.

### 3.2. Modeling Assumptions

The mathematical model is developed based on the following assumptions:

(1) The system operates in a consistent, steady-state condition;
(2) Heat losses from the components and connecting piping to the surroundings, as well as pressure drops along the piping, are ignored [20];
(3) The refrigerant liquid at the condenser outlet and the vapor from the evaporator are both saturated, whereas the vapor at the generator outlet is superheated;
(4) The solutions exiting the generator and absorber are saturated;
(5) The heat introduced into the system by the solution pump is neglected;
(6) The enthalpies of the solution remain unchanged before and after passing through the throttle;
(7) The ohmic resistance at both electrodes is ignored;
(8) Parasitic currents and concentration polarization effects are also neglected.

### 3.3. ARS Subsystem Model

Once the condenser and evaporator temperatures have been established, the corresponding pressures are derived using the following empirical equation:

(1) $$P(T)=P_{c}\exp\left[\frac{T_{c}}{T}\sum_{i=1}^{6}\alpha_{i}{\left(1-\frac{T}{T_{c}}\right)}^{\beta_{i}}\right]$$

where $P_{c}$ is the critical pressure of water, 22.064 × 10^6^ Pa; $T_{c}$ is the critical temperature of water, 647.096 K; and coefficients $\alpha_{i}$ and $\beta_{i}$ are given in Ref. [21]. *T* is the condensing or evaporating temperature.

Once the temperature and pressure of the absorber, evaporator, generator, and condenser have been accurately determined, the concentrations of the solute in both the concentrated and dilute solutions can be derived by [22]

(2) $$X=\sum_{0}^{3}Ant_{l}^{n}+t\sum_{0}^{3}Bnt_{l}^{n}+t^{2}\sum_{0}^{4}Cnt_{l}^{n}+t^{3}\sum_{0}^{3}Dnt_{l}^{n}$$

where t is the temperature of the solution, °C; $t_{l}$ is the saturation temperature of water at a pressure of *P*, °C.

After determining the temperatures and pressures of the water exiting the generator, condenser, and evaporator, the enthalpy is subsequently calculated by

(3) $$h_{w}=h_{c}\left[1+\sum_{i=1}^{4}\alpha_{i}{\left(1-\frac{T}{T_{c}}\right)}^{\beta_{i}}\right]$$

where $h_{c}$ is the specific enthalpy of pure water at the critical point, $37,548.5\;J\cdot \mathrm{mol}\cdot K^{-1}$; the coefficients are given in Ref. [21].

Taking assumption (6) into account, the energy equation pertaining to the throttling device can be streamlined and simplified accordingly by [22]

(4) $$h_{8}=h_{9}$$

where *h* is the enthalpy of the refrigerant water, $J\cdot \mathrm{mol}\cdot K^{-1}$.

The determination of the enthalpy of the solution at the absorber and generator outlets is based on the temperature and concentration:

(5) $$h_{s}(x,T)=(1-x_{molar})h_{w}+h_{c}\sum_{i=1}^{30}a_{i}x_{molar}^{m_{i}}{\left(0.4-x_{molar}\right)}^{n_{i}}{\left(\frac{T_{c}}{T-T_{0}}\right)}^{t_{i}}$$

where x and $x_{molar}$ are the mass and molar concentrations of the lithium bromide solution, respectively; $T_{0}$ is the reference temperature, which is 221 K; the values of coefficients $a_{i}$, $m_{i}$, $n_{i}$, and $t_{i}$ in the equation are given in Ref. [21].

Given assumptions (1) and (2), once the heat load of the evaporator and the refrigerant parameters at both the inlet and outlet have been determined, the refrigerant flow rate can be derived by applying the energy balance equation [22]:

(6) $${\dot{m}}_{r}=\frac{{\dot{Q}}_{e}}{h_{10}-h_{9}}$$

The mass flow rates of both the dilute and concentrated solutions can be deduced through the mass balance equation of the absorber [22]:

(7) $${\dot{m}}_{6}=\frac{{\dot{m}}_{r}X_{1}}{X_{6}-X_{1}}$$

(8) $${\dot{m}}_{1}=\frac{{\dot{m}}_{r}X_{6}}{X_{6}-X_{1}}$$

Next, we compute the heat capacities of the solution at state points 2 and 4 and ascertain the temperatures of the dilute solution as it enters the generator (state point 3) and the concentrated solution as it enters the absorber, using the energy-control equation related to the solution heat exchanger:

(9) $$c_{p,s}(T,x)=(1-x_{molar})c_{p,w}(T)+c_{p,t}\sum_{i=1}^{8}a_{i}x_{molar}^{m_{i}}(0.4-x_{molar})n_{i}(\frac{T_{c}}{T-T_{0}}{)}^{t_{i}}$$

where $c_{p,t}=76.0226\;J\cdot \mathrm{mol}\cdot K^{-1}$, $T_{t}$ = 273.16 K, and $m_{i}$, $n_{i}$, $t_{i}$, and $a_{i}$ are given in Ref. [21].

The temperatures and enthalpies of the solutions both entering and exiting the solution heat exchanger can be derived from the energy balance equation governing the solution heat exchanger [6].

When ${\dot{m}}_{4}c_{p4}<{\dot{m}}_{2}c_{p2}$,

(10) $$t_{5}=t_{4}-\varepsilon (t_{4}-t_{2})$$

When ${\dot{m}}_{4}c_{p4}\geq {\dot{m}}_{2}c_{p2}$,

(11) $$t_{3}=t_{2}-\varepsilon (t_{4}-t_{2})$$

(12) $${\dot{m}}_{4}h_{4}+{\dot{m}}_{2}h_{2}={\dot{m}}_{3}h_{3}+{\dot{m}}_{5}h_{5}$$

The enthalpy of the solution before and after passing through the throttling device is determined based on the principle of energy balance:

(13) $$h_{5}=h_{6}$$

The heat transfer capacities of the absorber, generator, and condenser are calculated based on the energy balance of each respective component [22]:

(14) $${\dot{Q}}_{a}={\dot{m}}_{r}h_{10}+{\dot{m}}_{6}h_{6}-{\dot{m}}_{1}h_{1}$$

(15) $${\dot{Q}}_{g,ARS}={\dot{m}}_{r}h_{7}+{\dot{m}}_{6}h_{4}-{\dot{m}}_{1}h_{3}$$

(16) $${\dot{Q}}_{g,RED}={\dot{m}}_{18}h_{18}+{\dot{m}}_{15}h_{15}-{\dot{m}}_{14}h_{14}$$

(17) $${\dot{Q}}_{c}={\dot{m}}_{r}(h_{7}-h_{8})$$

The coefficient of performance (COP) of the refrigeration system is calculated by

(18) $$\mathrm{COP}=\frac{{\dot{Q}}_{e}}{{\dot{Q}}_{g,ARS}}$$

### 3.4. RED Subsystem Model

The voltage producible by each IEM pair is computed using the Nernst equation [23]:

(19) $$E_{cell}(x)=(\alpha_{AEM}+\alpha_{CEM})\frac{RT}{zF}\ln(\frac{\gamma_{HC}C_{HC}}{\gamma_{LC}C_{LC}})$$

where α is the selective permeability coefficient of the IEM; z is the ionic valence; F is Faraday’s constant; γ is the ionic activity coefficient; C is the concentration of the solution, mol/L; the subscripts AEM and CEM denote the anion and cation exchange membranes; and HC and LC denote the concentrated and dilute solutions feeding the RED stack.

The resistance of both the concentrated and dilute solutions within each compartment inside the RED stack can be derived from Ohm’s law.

(20) $$r_{HC}(x)=f_{y}\frac{\delta_{HC}}{\kappa_{HC}(x)}$$

(21) $$r_{LC}(x)=f_{y}\frac{\delta_{LC}}{\kappa_{LC}(x)}$$

where $f_{y}$ is the shading coefficient; δ is the thickness of the solution compartment, m; and κ is the solution conductivity.

The ohmic resistance of the RED stacks is composed of the resistance of both the concentrated and dilute solutions, along with the resistance of the anion and cation exchange membranes, and the electrode resistance.

(22) $$R(x)=N(R_{LC}+R_{HC}+R_{AEM}+R_{CEM})+R_{el}$$

where $R_{el}$ is the electrode resistance; N is the number of IEMs.

The open-circuit voltage (*OCV*) is the voltage generated by the RED stack when the current is zero:

(23) $$OCV=NE_{cell}(x)$$

When the current is not zero, the output voltage is as follows:

(24) $$E_{stack}(x)=OCV-R(x)j(x)$$

where j(x) is the current density at position *x*.

The intensity of the current density is directly correlated to the load applied to the RED stack, which can be accurately determined through the application of Ohm’s law.

(25) $$j(x)=\frac{E_{stack}(x)}{R(x)}$$

The power density is calculated by

(26) $$P_{d}(x)=j(x)E_{stack}(x)$$

In the case of an ideal 100% selectively permeable IEM, only counter ions—those bearing the opposite charge to the fixed ions within the IEM—are permitted to traverse each membrane. However, a practical IEM is unable to fully reject these ions [24]. Consequently, the total salt flux can be articulated as follows [25]:

(27) $$J_{tot}(x)=\frac{j(x)}{F}+\frac{2D_{salt}}{\delta_{m}}\left[C_{HC}(x)-C_{LC}(x)\right]$$

The concentration-driven water flux is calculated by

(28) $$J_{osm}(x)=-2L_{p}vRT\left[\varphi_{HC}C_{HC}(x)-\varphi_{LC}C_{LC}(x)\right]$$

The electro-osmotic flux of water due to ion transport across the membrane is

(29) $$J_{esom}(x)=n_{h}\cdot J_{tot}(x)$$

The net flux of water is calculated as

(30) $$J_{w}(x)=J_{osm}(x)+J_{eosm}(x)$$

The concentration changes of the concentrated or dilute solution along the direction of flow of the solution due to the migration of water and ions through the IME are [26]

(31) $$\frac{dC_{HC}(x)}{dx}=\frac{b}{V_{HC}}J_{tot}(x)+\frac{b}{V_{HC}}\cdot C_{HC}(x)\cdot J_{w}(x)\cdot \rho_{w}$$

(32) $$\frac{dC_{LC}(x)}{dx}=\frac{b}{V_{LC}}J_{tot}(x)+\frac{b}{V_{LC}}\cdot C_{LC}(x)\cdot J_{w}(x)\cdot \rho_{w}$$

The Gibbs free energy between the concentrated and dilute solutions is calculated by [27].

(33) $$\Delta G=2RT(V_{HC}C_{HC}\ln\frac{C_{HC}}{C_{m}}+V_{LC}C_{LC}\ln\frac{C_{LC}}{C_{m}})$$

The efficiency of the RED stacks can be calculated by

(34) $$\eta_{RED}=P/\Delta G$$

The energy efficiency of a RED heat engine can be expressed as follows:

(35) η=P/Q

where Q represents the heat load of the RED generating unit, W.

The energy efficiency of the RED–ARS integration system is defined as

(36) $$\eta_{total}=\frac{Q_{e}+P}{Q_{g,ARS}+Q_{g,RED}}$$

The efficiency of a Carnot heat engine is calculated as

(37) $$\eta_{c}=1-T_{h}/T_{c}$$

where $T_{h}$ is the temperature of the heat source, °C; $T_{c}$ is the temperature of the heat sink, °C.

The thermodynamic perfectibility $\eta_{s}$ is calculated by

(38) $$\eta_{s}=\eta_{t}/\eta_{c}$$

where $\eta_{t}$ is

(39) $$\eta_{t}=\Delta G/Q_{g,RED}$$

### 3.5. Model Validation

**Figure 3:**
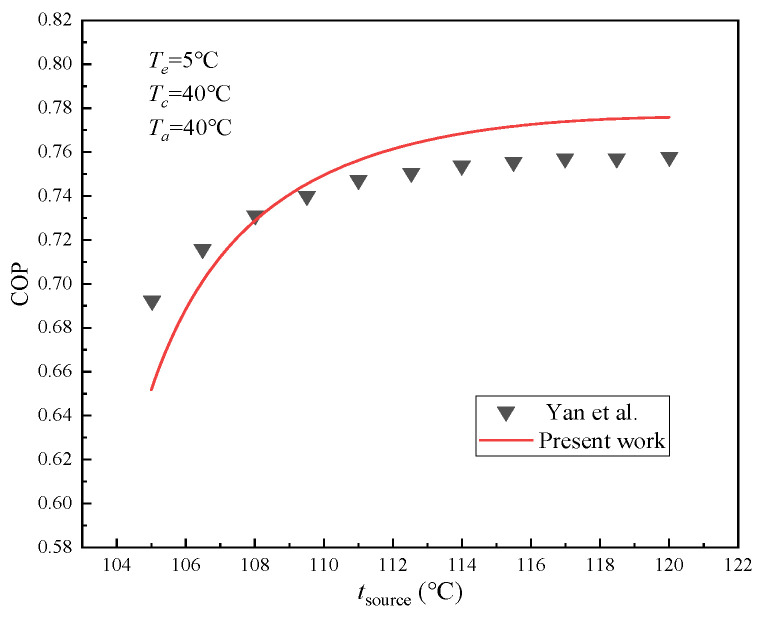
Comparison of ARS simulation modeling results with literature data [5].

The calculated results obtained from the established ARS mathematical simulation model were meticulously compared with data sourced from the literature [5] to certify its accuracy, as demonstrated in Figure 3. The input parameters were set as follows: the evaporation temperature was 5 °C, the condensation temperature was 40 °C, the absorber temperature was 40 °C, the heat transfer temperature difference was 10 °C in the solution heat exchanger, and the temperature difference between the heat source and generator was 20 °C.

**Figure 4:**
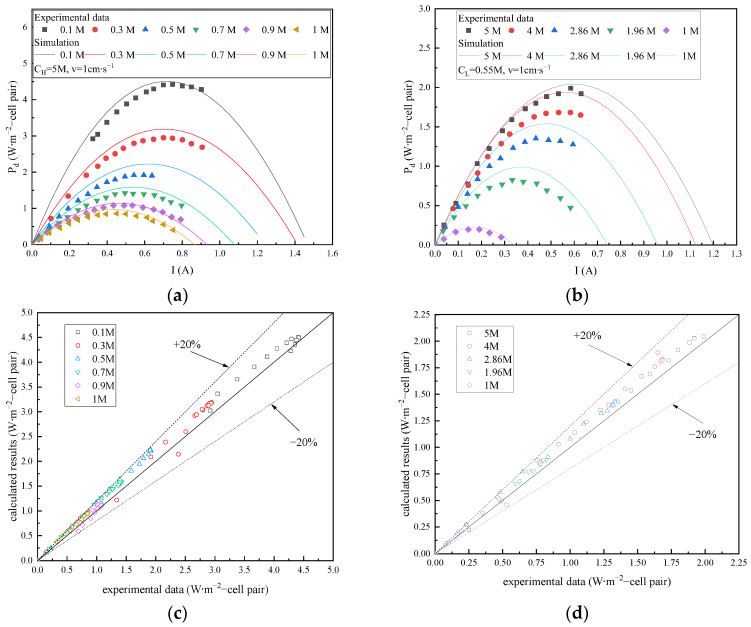
Comparison of the RED experimental and numerical calculation results: (**a**) change in concentration of dilute solution; (**b**) concentration change in concentrated solution; (**c**) deviation in the experimental data and calculated results under variations in the concentration of the dilute solution; (**d**) deviation in the experimental data and calculated results under variations in the concentration of the concentrated solution.

The RED mathematical simulation outcomes of this study were compared with experimental data obtained by Tedesco et al. [25], as shown in Figure 4. By adhering to the experimental conditions, a single RED stack employed 50 IEM pairs, each of which measured 0.1 × 0.1 m^2^, and the thickness of the solution compartment was 270 μm. The working solution consisted of an aqueous NaCl solution maintained at a temperature of 25 °C and the flow rate of 1 cm∙s^−1^. The concentrations of the concentrated and dilute solutions were independently varied, and the resultant output power densities of the RED stacks were calculated and subsequently compared with the test values reported in the literature.

### 3.6. Operational Strategy and Methodology

**Figure 5:**
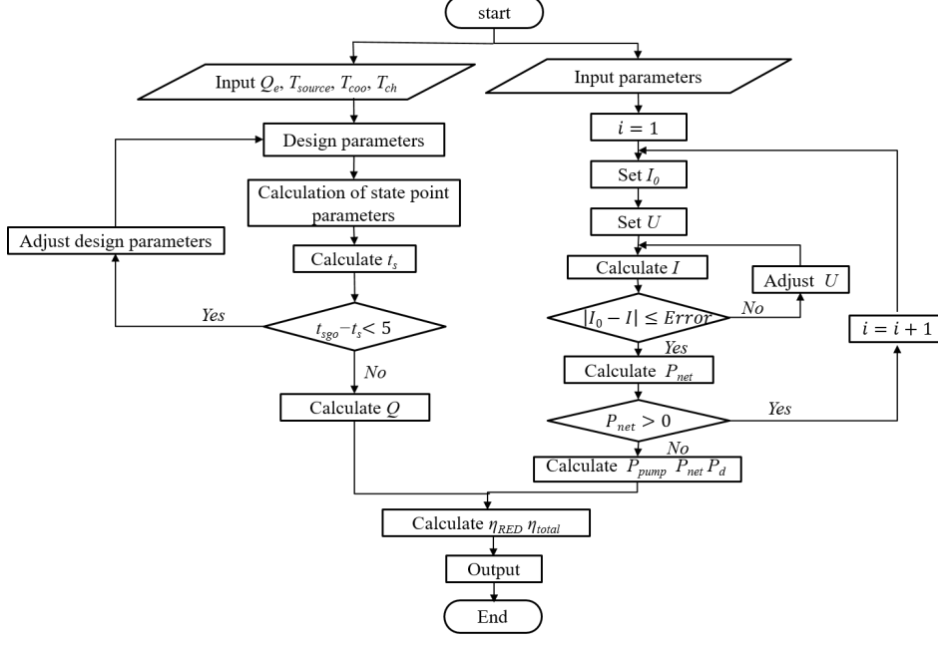
The flow chart of the simulation model of a RED–ARS integration system.

The comprehensive mathematical simulation model for the RED–ARS integration system was programmed in the computational flowchart, as depicted in Figure 5. On the left side, the flowchart illustrates the computational process of the ARS subsystem. This subsystem is responsible for generating cold capacity and regenerating the concentrations of the working solutions, ultimately providing the feed solutions for the RED stacks. The right side details the computational process of the RED subsystem, which is terminated when the output power of the last RED stack falls below zero.

## 4. Discussion

### 4.1. Effect of Heat Source Temperature

The driving heat source of the generator—specifically for the low-grade waste heat—exhibits instability in the actual operation of the unit. The temperature fluctuations of the heat source directly impact the unit’s performance. An increase in the generation temperature leads to a heightened concentration of the concentrated solution, thereby enhancing the SGE between the concentrated and dilute solutions (C_HC_ and C_LC_). The modulation of the heat source temperature alters the generation temperature of the working solution. Additionally, it considers the influence of the solution’s crystallization. When the generation temperature surpasses a threshold, resulting in an excessively high concentration of the solution, there is a risk of surpassing its crystallization concentration. Such an occurrence triggers crystallization issues, ultimately compromising the unit’s performance [28].

In this study, the impact of the heat source temperature on the performance parameters associated with the RED–ARS integration system was examined within a temperature range of 90 °C to 130 °C. The condensing temperature of the dilute solution, C_HC_, C_LC_, the flow rate, and the solution temperature in the RED stacks were maintained at 70 °C, 5 mol∙L⁻^1^, 0.02 mol∙L⁻^1^, 1 cm∙s⁻^1^, and 40 °C, respectively. The ARS subsystem’s cooling capacity was set at 70 kW, the evaporation temperature was 5 °C, and the condensation temperature was 40 °C. Additionally, the dilute solution concentration, flow rate, and solution temperature in the RED stacks were controlled at 0.02 mol∙L⁻^1^, 1 cm∙s⁻^1^, and 40 °C, respectively.

**Figure 6:**
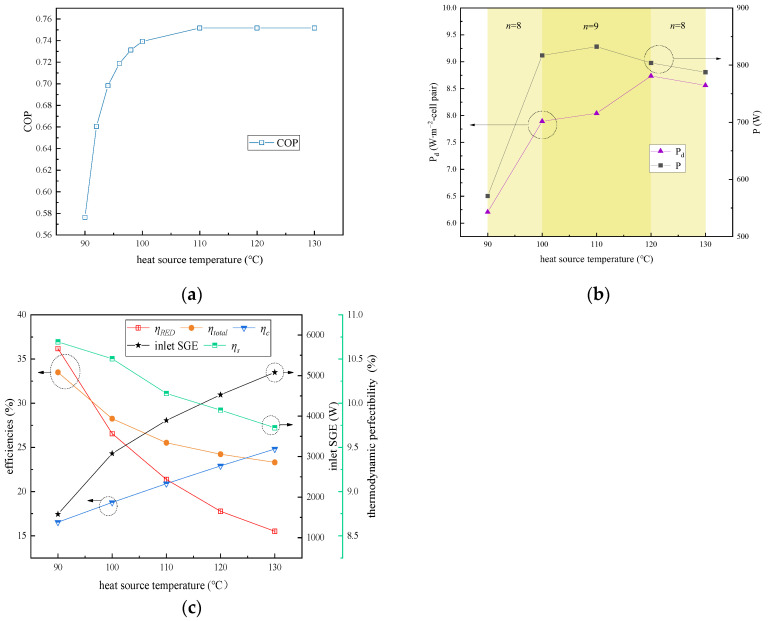
Effect of heat source temperature on RED–ARS system performance: (**a**) change in COP; (**b**) change in output power and power density; (**c**) change in inlet SGE and energy efficiencies.

As illustrated in Figure 6a, the COP of the ARS gradually rises with an increase in the heat source temperature until it is stabilized. This is attributed to the fact that the elevation of the heat source temperature can lead to an increase in the generation temperature of the ARS generator unit. Consequently, the solution concentration at the outlet of the generator is increased, resulting from the separation of more refrigerant water vapors from the solution. Figure 6b demonstrates that the output power initially increased but then decreased with an increase in the heat source temperature. The observed declining trend at high heat source temperatures is attributed to the fact that a higher concentration of the HC solution, separated from the ARS generator unit, may lead to a decrease in the selectivity of the IEMs within the RED stacks.

As Figure 6c shows, the Carnot efficiency of the thermal separation system also increases as the heat source temperature increased. However, the thermodynamic perfectibility decreases with a rising heat source temperature, indicating an increase in the system’s degree of irreversibility, related to the enlargement of the heat transfer temperature difference.

### 4.2. Effect of Condensing Temperature of Dilute Solution in RED Stacks

**Figure 7:**
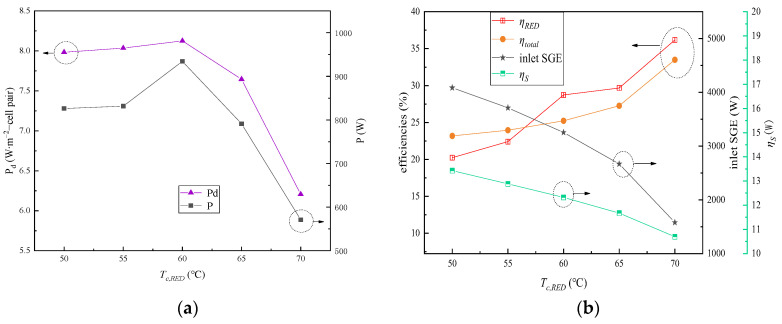
Impact of the condensing temperature of the dilute solution in the RED stack variation on the RED–ARS system performance: (**a**) changes in output power and power density; (**b**) changes in inlet SGE and energy conversion efficiencies.

The impact of the condensing temperature of the dilute solution (*T_c,RED_*) in the RED stacks when it was varied from 50 °C to 70 °C on the output power, power density, inlet SGE, and energy efficiency of the RED–ARS system was examined and is depicted in Figure 7. During the investigation, the heat source temperature, C_HC_, C_LC_, the flow rate, and the solution temperature in the RED stacks were maintained at 90 °C, 5 mol∙L⁻^1^, 0.02 mol∙L⁻^1^, 1 cm∙s⁻^1^, and 140 °C, respectively.

As illustrated in Figure 7a, both the output power and power density exhibited an initial increase followed by a decrease as *T_c,RED_* decreased. Meanwhile, the RED efficiency (*η_RED_*) and the total system efficiency (*η_total_*) gradually increased with the increase in *T_c,RED_*, as seen in Figure 7b. Conversely, the thermodynamic perfectibility of the RED generating unit gradually improved. This trend was attributed to the increase in the condensation pressure caused by *T_c,RED_*. The efficiency of the Carnot heat engine is solely dependent on the temperatures of the heat source and heat sink; thus, *η_s_* gradually decreases with the increase in *T_c,RED_*.

When *T_c,RED_* is reduced, the generator’s effectiveness can be increased, thus generating more water vapor and elevating the concentration of the concentrated solution at the generator outlet. This, in turn, augments the SGE between the feed solutions. It further results in an initial increase in the output power of the RED stacks. The increase in the concentrated solution’s concentration enhances the membrane potential, which is beneficial to increase the *OCV* of the RED stack. However, as the HC solution concentration increases, the permselectivity of the membrane gradually decreases. This leads to a gradual decline in the output power and a reduction in the power density after *T_c,RED_* reaches 60 °C. Since the rate of increase in SGE with decreasing *T_c,RED_* is greater than the rate of increase in the output power, *η_RED_* gradually decreases as *T_c,RED_* declines. On the other hand, the enhancement in the generation effect and the increase in separation efficiency with decreasing *T_c,RED_* lead to a gradual increase in the system’s efficiency.

### 4.3. Effect of Salinity Gradients

The concentrations of both the feed concentrated and dilute solutions have a direct impact on the SGE, electromotive force, ohmic resistance, and, ultimately, the output performance of the RED stacks and the energy efficiency of the whole system.

**Figure 8:**
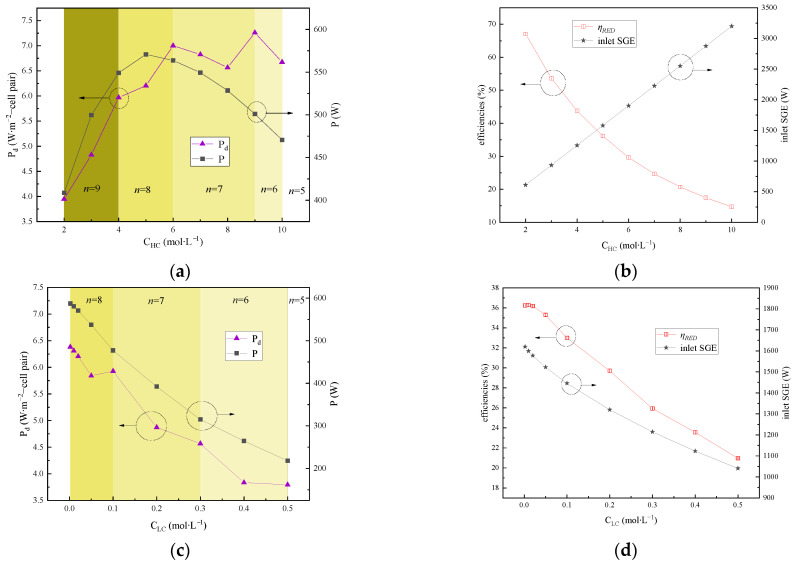
Impact of the concentration gradient variation on the system performance: (**a**) impact of C_HC_ on output power and power density; (**b**) impact of C_HC_ on inlet SGE and energy efficiency; (**c**) impact of C_LC_ on output power and power density; (**d**) impact of C_LC_ on SGE and energy efficiency.

Figure 8a,b depict the impacts of varying C_HC_ on the output power and efficiency. Either the output power or power density exhibits a parabolic-type variation phenomenon (increases initially, followed by a decrease) when C_HC_ increases from 3 mol∙L⁻^1^ to 9 mol∙L⁻^1^. This trend is in accordance with the increase–decline variation in the average ionic activity coefficient of the aqueous LiBr solution with the continuing increase in the solution concentration [29,30]. Furthermore, the elevated C_HC_ enhances the salinity gradient, resulting in augmented ionic migration flux in the concentrated solution and increased water permeation flux in the dilute solution. As a consequence, this escalates the SGE loss. Additionally, the selective permeability coefficient of the IEM diminishes with the rising concentration of the aqueous LiBr solution [31]. This ultimately results in the observed variation trend.

*η_RED_* progressively decreases with rising C_HC_. This is because the increase in the input SGE is more pronounced compared to the output electrical energy with the increase in C_HC_. Thus, as per Equation (34), it leads to a decline in RED efficiency. It is crucial to maintain an appropriate concentration of the concentrated solution, as excessively high lithium bromide concentrations at a given temperature can pose crystallization challenges, as well as resulting in output performance attenuation.

The impact of variations in the C_LC_ concentrations, ranging from 0.002 mol∙L^−1^ to 0.5 mol∙L^−1^, on both the output power and efficiency were examined, as depicted in Figure 8c,d. In this investigation, the C_HC_ concentration, flow rate, and solution temperature in the RED stacks were maintained at 5 mol∙L^−1^, 1 cm∙s^−1^, and 40 °C, respectively. Figure 8c reveals clearly that, as C_LC_ increases, both the output power and power density exhibit a gradual decline. Notably, when C_LC_ is 0.1 mol∙L^−1^, the number of RED stacks that can be interconnected in series decreases from *n* = 8 to *n* = 7, leading to a marginal enhancement in the power density at this specific concentration. This phenomenon can be attributed to the accelerated increase in the concentration of the dilute solution owing to the ionic increase due to C_HC_. Consequently, the salinity gradient between the two solutions diminishes at a more rapid pace, leading to a swift decrease in the SGE. As Equation (19) describes, the electromotive force decreases with rising C_LC_, ultimately causing a reduction in both the output power and power density of the RED stacks.

Furthermore, although an increase in C_LC_ leads to an enhancement in the solution conductivity, thereby decreasing the resistance of the dilute solution compartment, the concurrent decrease in the salinity gradient with rising C_LC_ thereby diminishes the driving force for ionic migration. This, it results in a gradual decrement in the inlet SGE of the RED stacks, as shown in Figure 8d.

Regarding *η_RED_*, Figure 8d demonstrates a slight initial increase but this is rapidly followed by a gradual decrease with rising C_LC_. Initially, the significant rise in the conductivity of the dilute solution decreases its resistance, causing the rate of decrease in the output power to be smaller than the rate of decrease in the inlet SGE. This results in the elevation of *η_RED_* in a short range. However, as C_LC_ continues to increase, the decrement in the output power surpasses the decrement in SGE, leading to a progressive decline in *η_RED_* with increasing C_LC_.

### 4.4. Effect of Number of RED Stacks in Multi-Stage System

If only a single RED stack is used, there is a significant portion of SGE that remains unused in the effluent at the outlet of the RED stack, posing challenges in achieving higher energy conversion efficiency and hindering its practical implementation in LTE recovery. This section explores the effects of varying the number of RED stacks in a multi-stage system on both the output power and energy efficiency, across different salinity gradient scenarios.

**Figure 9:**
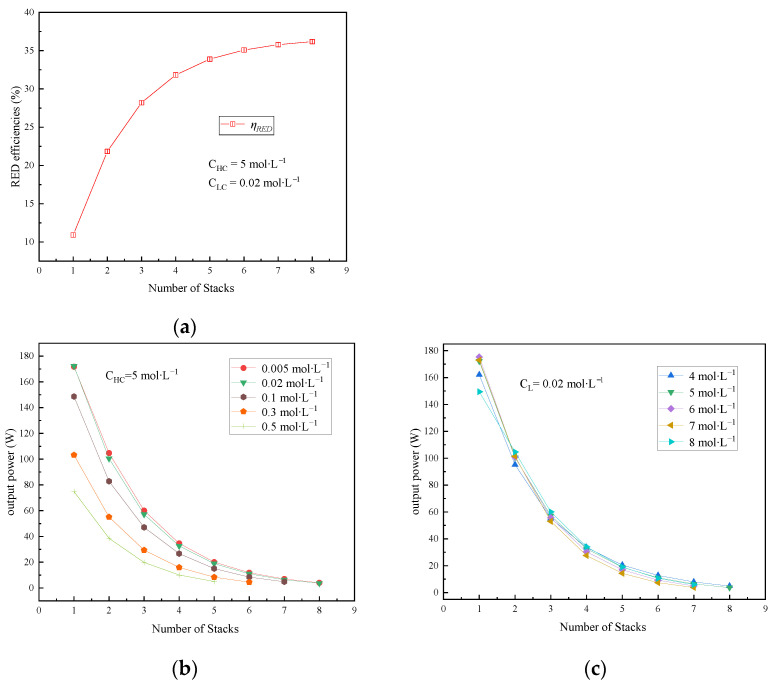
Impact of the number of RED stacks in series system on performance: (**a**) impact on energy efficiency; (**b**) impact on output power under varying C_LC_; (**c**) impact on output power under varying C_HC_.

As Figure 9a illustrates, *η_RED_* gradually rises (with a slow acceleration rate) as the number of RED stacks increases at the feed solution conditions in which C_HC_ is 5 mol∙L^−1^ and C_LC_ is 0.02 mol∙L^−1^. As Figure 9b,c show, the output power of each individual RED stack exhibits a gradual decline with an increase in the number of stacks. This is attributed to the reduction in the available energy reserved in the HC and LC solutions in the multi-stage series RED system. It progressively diminishes with an increase in the RED stack number; ultimately, the output power approaches zero. Meanwhile, Figure 9c reveals an intriguing trend: the output power of the first RED stack initially increases and then decreases with an elevation in C_HC_. When the *C_LC_* is constant, the electromotive force increases with increasing *C_HC_*. At the same time, the membrane permeation of salt becomes more severe with increasing *C_HC_* in Equation (27), leading to an increase in the irreversible loss of SGE. Therefore, the first stack reaches the optimum value of power output when the concentration of the concentrated solution is 6 mol∙L⁻^1^. The electromotive force of the RED stacks in the series system becomes sequentially lower due to the gradual decrease in SGE between the HC and LC solutions flowing through multiple RED stacks.

### 4.5. Effect of Flow Rate

The flow rate plays a pivotal role in determining the residence time of the working solution within the RED stacks, thereby influencing both the water flux and ionic flux across the IEM. This section explores the effects of varying flow rates, ranging from 0.4 cm∙s⁻^1^ to 1.3 cm∙s⁻^1^, on the output power and energy efficiency. The C_HC_, C_LC_, and solution temperature in the RED stacks were held constant at 5 mol∙L⁻^1^, 0.02 mol∙L⁻^1^, and 40 °C, respectively.

**Figure 10:**
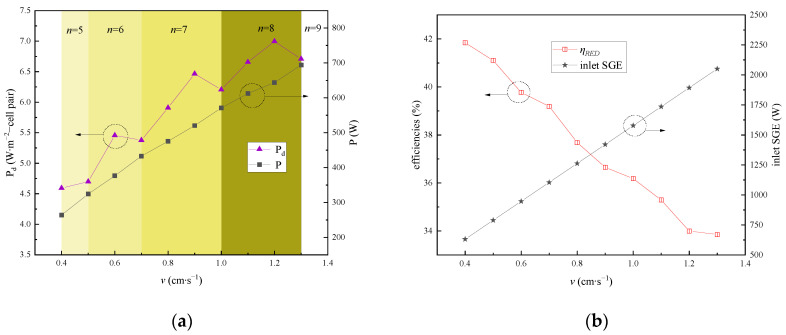
Effects of the solution flow rate variation in the RED stack on system performance: (**a**) impact on output power and power density; (**b**) impact on inlet SGE and energy conversion efficiency.

As Figure 10a illustrates, with the increase in the flow rate, both the output power and power density gradually rise. Notably, as the number of RED stacks increases with the flow rate, an inflection point in the power density emerges during this upward trend. Conversely, Figure 10b shows a gradual decline in RED efficiency with an increase in the solution flow rate. The underlying reason for these observations is as follows: as the flow rate intensifies, the residence time of both the concentrated and dilute solutions in their respective compartments diminishes. Consequently, the concentration attenuation of the working solution on both sides of the IEM decreases in the flow direction. This results in a reduced variation in the SGE along the flow path, leading to an increase in the potential available energy for the subsequent RED stacks. This allows for the connection of more RED stacks in the multi-stage system, thereby generating more electric energy in total. The enhanced flow rate also boosts the flow of the concentrated and dilute solutions at the entrance of the stacks. Consequently, as depicted in Figure 10b, the SGE at the entrance of the RED stacks gradually increases with an increase in the solution flow rate across the membrane.

To a certain extent, a lower flow rate permits the salt ions in the solution to reside for longer within the flow channels of the RED stacks, affording more time for mass transfer and facilitating the more efficient exploitation of the SGE. Consequently, a greater proportion of this energy can be converted into electrical energy. However, an excessively low flow rate tends to exacerbate the concentration polarization on both sides of the IEM, leading to increased potential loss due to boundary layer resistance. Furthermore, while increasing the flow rate can indeed result in higher output power, in practical applications, an excessively high flow rate can cause the solution’s flow flux and flow resistance to surge. This instability can affect the membrane and elevate the power consumption of the pump. Therefore, it is crucial to maintain a reasonably controlled solution flow rate.

### 4.6. Effect of Solution Temperature in the RED Stacks

**Figure 11:**
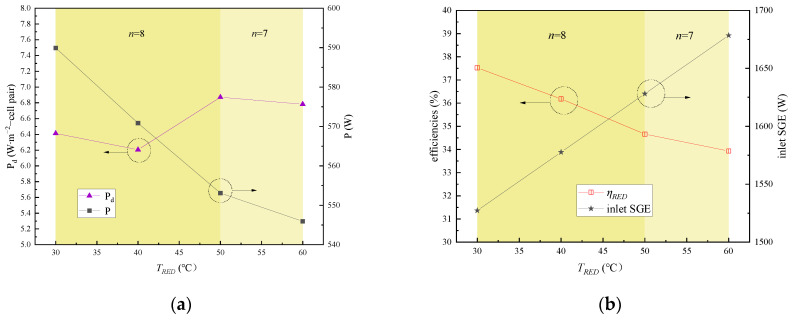
Impact of solution temperature variation in RED stack on system performance: (**a**) impact on output power and power density; (**b**) impact on SGE and energy efficiency.

The impact of solution temperature variations in the RED stacks was examined, ranging from 30 °C to 60 °C, regarding the output power and energy efficiency. During this section, C_HC_, C_LC_, and the flow rate were maintained at 5 mol∙L⁻^1^, 0.02 mol∙L⁻^1^, and 1 cm∙s⁻^1^, respectively. As depicted in Figure 11a, the output power exhibits a gradual decline with an increase in the solution temperature in the RED stacks. Specifically, the power density undergoes an initial dip at 50 °C due to a reduction in the number of RED stacks in the multi-stage system. This trend persists as the solution temperature in the RED stacks continues to rise. However, it is worth noting that the power density briefly increases at 50 °C before resuming its downward trajectory; this is also related to the number of practical connected RED stacks in the whole system in series. The following is a detailed explanation: according to Equation (19), the OCV increases with the temperature, and so does the solution conductivity. Consequently, the resistances of both the concentrated and dilute solutions decrease. While this leads to an initial boost in the output power of the first-stage RED stack, the residual SGE available for subsequent stacks diminishes. As a result, as the solution temperature in the RED stacks increases, and the output power of each subsequent series of stacks diminishes, limiting the total number of connectable stacks. Ultimately, this combination of factors contributes to a decline in the total output power with the rising solution temperature in the RED stacks.

Conversely, as shown in Figure 11b, increasing the solution temperature in the RED stacks leads to an increase of the inlet SGE, as predicted by Equation (33). Despite this, as Equation (34) shows, the growth rate of the power generation is less than that of the SGE, and thus *η_RED_* gradually decreases with the increasing solution temperature in the RED stacks, as depicted in Figure 11b.

## 5. Conclusions

In this paper, a comprehensive mathematical model for the RED–ARS integration system is presented. This system harnesses LGTE to convert the SGE of LiBr solutions with different concentrations into electrical energy. On this basis, the authors simulated and discussed the impact of various factors on the performance of the RED–ARS integration system. These factors included the heat source temperature, concentrated solution concentration, dilute solution concentration, condensing temperature of the dilute solution, solution temperature and flow rate in the RED stacks, and the number of RED stacks. The conclusions are drawn as follows.

- The output power initially rises and subsequently declines as the heat source temperature (ranging from 90 to 130 °C), *T_c,RED_* (50 to 70 °C), and C_HC_ (3 to 9 mol∙L⁻^1^) increase. Moreover, it decreases with an increase in C_LC_ (0.002 to 0.5 mol∙L⁻^1^) and an increasing solution temperature in the RED stacks. However, it shows an upward trend with an increase in *v* (0.4 to 1.3 cm∙s⁻^1^). The peak output power achieved is 934 W.
- The coefficient of performance (COP) of the ARS escalates as the heat source temperature rises, achieving a maximum COP of 0.75.
- The heat source temperature, *T_c,RED_*, C_HC_, C_LC_, flow rate (*v*), and *T_RED_* all exert an influence on the energy efficiency of the RED–ARS integration system. The theoretically optimal values for these parameters are determined to be 90 °C, 70 °C, 6 mol∙L⁻^1^, 0.002 mol∙L⁻^1^, 0.4 cm∙s⁻^1^, and 40 °C, respectively. Under these conditions, the maximum energy efficiency of 33% is attained.
- The comprehensive mathematical model of the RED–ARS integration system facilitates the simulation of various aspects of energy conversion performance, including the cooling capacity, SGE to electricity, and LGTE to SGE and then to electricity. This model enables the determination of the optimal operating conditions and parameters, thereby providing valuable reference data for practical applications.
- In practice, by judiciously enhancing the heat exchange area of the system, it becomes fully operational in areas where electricity is in short supply or where there is no power grid whatsoever.

## Figures and Tables

**Table 1:** Modeling of structural and operational parameters.

Item	Parameter	Symbol	Value
ARS	cooling capacity	*Q_e_*	70 kW
	temperature of cooling water inlet and outlet	*T_co_*	30 °C/37 °C
	temperature of chilled water inlet and outlet	*T_ch_*	14 °C/7 °C
RED	number of cell pairs	*N*	230
	solution flow compartment 1	*l* × *b* × *δ_HC_*	0.1 m × 0.5 m × 200 μm
	solution flow compartment 2	*l* × *b* × *δ_LC_*	0.1 m × 0.5 m × 100 μm
	membrane thickness	*δ_AEM_/δ_CEM_*	125 μm/135 μm

## Data Availability

The data presented in this study are available on request from the corresponding author.

## Nomenclature

**Nomenclature**		*X*	LiBr solution mass concentration, kg/kg
*Symbols*		*z*	Valence of ions
*b*	Width of the solution compartments in an RED cell unit	*Greek letters*	
*C*	Concentration, mol·L^−1^	*δ*	Thickness, m
*c_p_*	Specific heat, J·mol^−1^·K^−1^	*α*	Permselectivity of membrane
*D_salt_*	Salt permeability coefficient	*γ*	Activity coefficient
*E*	Voltage, V	Δ*G*	Maximum available SGE, W
*F*	Faraday constant, C·mol^−1^	*δ_m_*	Average membrane thickness, m
*f_y_*	Spacer shadow factor	*η*	Energy efficiency
*h*	Specific enthalpy, J∙kg^−1^	*κ*	Electrical conductivity, S·m^2^·mol^−1^
*I*	Current, A	*ρ*	Density, kg·m^−3^
*j*	Current density, A·m^−2^	*φ*	Osmotic coefficient
*J_eosm_*	Water flux carried by ion transmission, mol·m^−2^·s^−1^	*Superscripts and subscripts*	
*J_osm_*	Water flux driven by concentration	*a*	Absorber
	gradient, mol·m^−2^·s^−1^	*c*	Condenser
*J_tot_*	Salt flux, mol·m^−2^·s^−1^	*coo*	Cooling water
*l*	Length of the solution compartments in a RED cell unit, m	*ch*	Chilled water
		*g*	Generator
*L_p_*	Water permeability coefficient, m·Pa^−1^·s^−1^	*e*	Evaporator
*m*	Mass flow rate, kg·s^−1^	*HC*	Concentrated solution
M	Molarity, mol·L^−1^	*LC*	Dilute solution
*N*	Number of cell pairs	*mixed*	Mixed solution
*n*	Number of RED stacks	*w*	Water
*P*(*T*)	Pressure, Pa	*s*	Crystallization
*P*	Gross output power, W	*sgo*	Heat exchanger outlet concentrated solution
*P_d_*	Power density, W·m^−2^-cell pair		
*Q*	Heat transfer rate, W	*AEM*	Anionic exchange membrane
*R*	Universal gas constant, J·mol^−1^·K^−1^	*CEM*	Cationic exchange membrane
*r*	Electrical resistance, Ω·m^−2^	*ARS*	ARS subsystem
*T*	Temperature, K	*RED*	RED subsystem
*t*	Temperature, °C	*cell*	Cell pair
*E_stack_*	Output voltage of the stack, V	*el*	Electrode
